# Modeling the Health and Economic Impact of Substandard and Falsified Medicines: A Review of Existing Models and Approaches

**DOI:** 10.4269/ajtmh.21-1133

**Published:** 2022-07-13

**Authors:** Sachiko Ozawa, Colleen R. Higgins, Jude I. Nwokike, Souly Phanouvong

**Affiliations:** ^1^ Division of Practice Advancement and Clinical Education, UNC Eshelman School of Pharmacy, University of North Carolina, Chapel Hill, North Carolina;; ^2^ Department of Maternal and Child Health, UNC Gillings School of Global Public Health, University of North Carolina, Chapel Hill, North Carolina;; ^3^ Promoting the Quality of Medicines Plus (PQM+) Program, U.S. Pharmacopeial Convention, Rockville, Maryland

## Abstract

Substandard and falsified medicines are harmful to patients, causing prolonged illness, side effects, and preventable deaths. Moreover, they have an impact on the health system and society more broadly by leading to additional care, higher disease burden, productivity losses and loss of trust in health care. Models that estimate the health and economic impacts of substandard and falsified medicines can be useful for regulators to contextualize the problem and to make an economic case for solutions. Yet these models have not been systematically catalogued to date. We reviewed existing models that estimate the health and economic impact of substandard and falsified medicines to describe the varying modeling approaches and gaps in knowledge. We compared model characteristics, data sources, assumptions, and limitations. Seven models were identified. The models assessed the impact of antimalarial (*n* = 5) or antibiotic (*n* = 2) quality at a national (*n* = 4), regional (*n* = 2), or global (*n* = 1) level. Most models conducted uncertainty analysis and provided ranges around potential outcomes. We found that models are lacking for other medicines, few countries’ data have been analyzed, and capturing population heterogeneity remains a challenge. Providing the best estimates of the impact of substandard and falsified medicines on a level that is actionable for decision-makers is important. To enable this, research on the impact of substandard and falsified medicines should be expanded to more medicine types and classes and tailored to more countries that are affected, with greater specificity.

## INTRODUCTION

Approximately one in 10 essential medicines in low- and middle-income countries (LMICs) have been found to fail quality testing.^1^^,^^2^ The WHO defines these medicines as “substandard” when they do not meet their quality standards and/or specifications or “falsified” when they are intentionally made to misrepresent their identify, composition, or source.^3^ Patients who use substandard and falsified medicines can experience prolonged illness due to lack of or reduced effective active pharmaceutical ingredient, adverse effects due to toxic ingredients, and financial harm from having to pay for additional health care or needing to take time away from work. Yet such harm caused by substandard and falsified medicines is not fully understood, and the true impact on the population, health system, and economy is often unknown. As more data on the prevalence of substandard and falsified medicines are collected, efforts to estimate their health and economic consequences has become possible through decision-modeling. The results of these models can aid national medicines regulatory authorities (NMRAs) and health ministries to contextualize the issue alongside other public health investments and prioritize interventions that have a high return. This review compiles models used to estimate the health or economic burden attributable to substandard and falsified medicines to compare the mechanisms used for these estimations and to uncover gaps in knowledge within this field.

We provide a narrative overview of the literature comparing different modeling methods and synthesizing the findings. A search was performed in February 2021 in PubMed and Scopus to identify models that estimate the health and/or economic impact of substandard and falsified medicines. Search terms used were “substandard” or “falsified” and “medicines,” along with “model” or “impact.” Google was used to search for gray literature using the same search terms. Articles or standalone models were included if they used modeling techniques to predict or estimate the effects of substandard and/or falsified medicines across a population. Models that were already known to the authors were added to search results. We compared model approaches and characteristics, data sources, assumptions, and limitations.

## MODELS EXAMINING SUBSTANDARD AND FALSIFIED MEDICINES

Database searches resulted in 642 articles screened, including 375 articles in PubMed and 267 articles in Scopus. After screening each article, we identified seven models that fit our inclusion criteria. This included four models that estimated the burden of substandard and falsified medicines, two models that examined the cost-effectiveness of medicine quality screening technologies, and one model that examined the impact of poor-quality medicines on development of antimicrobial resistance. The seven models assessed the impact of poor-quality antimalarials (*n* = 5) or antibiotics (*n* = 2) and focused on estimating the impact at the national (*n* = 4), regional (*n* = 2), or global (*n* = 1) levels. Included were three decision tree models, two agent-based models, one statistical model, and one transmission dynamics model. Most models conducted uncertainty analysis and provided ranges around potential outcomes. Table 1 synthesizes the characteristics of each model.

**Table 1:** Characteristics of Models

	WHO/University of Edinburgh 2017^1^	WHO/London School of Hygiene and Tropical Medicine 2017^1^	Brock et al. 2017^12^	Luangasanatip et al. 2021^11^	SAFARI 2019–University of North Carolina^5^^–^^9^	ESTEEM 2021–University of North Carolina^10^	Renschler et al. 2015^4^
Purpose	Provided rough estimates of increased mortality of childhood pneumonia due to use of SF medicines	Modeled the health and economic cost of SF drugs for first-line treatment of uncomplicated *Plasmodium falciparum* malaria in sub-Saharan Africa	Modeled the health impact of poor-quality antimalarials on SP-resistant malaria infections	Estimated the cost-effectiveness of portable screening devices and 5-year budget impact of implementing them	Estimated the health and economic burden caused by SF antimalarials	Modeled SF prevalence on the market and the health and economic impact of quickly screening then removing SF amoxicillin before being used by patients	Estimated the number of under-5 malaria deaths attributable to poor-quality antimalarials
Scope	Global: broken into industrialized and developing countries	Regional: sub-Saharan Africa	National: Kenya	National: Laos	National: Benin, DRC, Uganda, Nigeria, Zambia	National: Kenya	Regional: sub-Saharan Africa
Medicine and population	Antibiotics used for pediatric ALRI/pneumonia	Antimalarials: ACTs and non-ACTs used for malaria, all ages	Antimalarials: SP and ACTs used for malaria, all ages	Antimalarials: ACTs used for all ages	Antimalarials: ACTs, chloroquine, quinine, monotherapy, used for pediatric malaria	Antibiotics: Amoxicillin used for pediatric pneumonia	Antimalarials: ACTs, SP used for pediatric malaria
Structure	Decision tree	Decision tree	Transmission dynamics model, with host and vector models	Decision tree	Agent-based model in which children are the agents	Agent-based model in which medicines are the agents	Statistical
Mechanism for modeling SF impact on patients	Twice the case fatality rate was applied for children using SF medicines Using the number of severe cases receiving treatment, estimated the number of deaths due to poor-quality antibiotics	SF medicines resulted in reduced effectiveness Baseline model was run with SF medicines and compared with scenario where all medicines were good quality	Substandard SP was modeled to act as if it were a reduced dose of SP, resulting in a lower rate of recovery in the human model and prolonged infectivity of malaria SF antimalarials were not modeled as directly affecting mortality. Malaria mortality was modeled to be related to malaria prevalence, which was impacted by the use of SF antimalarials.	SF antimalarials increased probability of severe disease and increased probability that severe cases result in death	SF medicines resulted in reduced effectiveness Baseline model was run with SF medicines and compared with scenario where all medicines were good quality	Twice the case fatality rate was applied for children using SF medicines Scenario implementing screening technology to quickly remove SF amoxicillin from the market was compared with scenarios where SF medicines were left on the market longer and used by patients	Increase in case fatality rate was applied for taking SF antimalarials
Main inputs & data sources	Deaths, severe cases, case fatality rate: previous modeling analysis specific to pediatric respiratory infection Hospital care for severe case: DHS and MICS on percentage of cases of suspected pneumonia that report receiving antibiotic treatment	Care-seeking: published literature, combined for all countries Disease prevalence: World Malaria Report & Clinton Health Access Initiative SF prevalence: published literature Costs: WHO CHOICE	Medicines received: DHS SF Prevalence: published literature, generalized to Kenya Gametocyte clearance when using SF (half-dose) antimalarials: calculated based on mice and human experimental data for use of SP	Cases: WHO Performance of the devices: laboratory experiments Costs: device costs from manufacturers, inspection costs from Laos FDD	Incidence/cases: World Malaria Report Care seeking: country-specific DHS and MIS surveys Medicines taken: country-specific MIS surveys SF Prevalence: published literature based on systematic review Costs: ACT Watch reports, published literature	Incidence/cases: published literature Care-seeking: published literature Case fatality rate: published literature Amoxicillin market breakdown: medicines sampling in Kenya SF Prevalence: medicines sampling literature in Kenya Costs: published literature	Malaria cases and care seeking: household surveys Private sector antimalarial sales: published literature Estimates for under-5 deaths and case fatality rates: WHO Global Health Observatory Data Repository SF Prevalence: published literature
Main Outputs	Number of excess deaths occurring due to SF antibiotics	Averted: Treatment failures Severe malaria cases Deaths DALYs Inpatient and outpatient costs	Number of SP resistant infections Total malaria cases Duration of gametocyte carriage	Costs of inspections using 6 screening technologies DALYs averted 5-year budget impact	Averted: Deaths Hospitalizations DALYs Cases of disability Direct costs Productivity losses	Costs of screening Averted: SF treatments Deaths Direct costs Productivity losses	Deaths caused by SF antimalarials
Uncertainty	Alternative scenario: 4 times the case fatality rate for those taking SF medicines Varied levels of SF prevalence at 1%, 5%, 10%	Univariate sensitivity analysis varied: Prevalence of SF Amount of efficacy reduction for SF medicines % patients that receive any antimalarial % of ACTs vs. non-ACTs % progressing to severe % receiving further inpatient or outpatient care CFR Costs of care	Univariate sensitivity analysis was run with min and max values Identified important parameters as: • Proportion of mosquitoes to humans • Daily rate mosquitoes reach adulthood • Probability of transmission of SP-sensitive and SP-resistant sporozoites during a blood meal • Gametocyte clearances of SP-sensitive/resistant gametocytes when treated with AL	Scenario analysis varied levels of SF medicines, sampling techniques (1, 2, or 3 samples tested per device), and number of devices implemented. One-way sensitivity analyses were conducted on costs, device performance, years of life lost to malaria	Probabilistic sensitivity analysis was run within model across more than 10,000 model runs • Beta distributions used for costs • Gamma distributions used for rates Policy scenarios were run to contextualize the burden of SF compared to other issues: lower stockouts, care seeking increases, perfect adherence, ACT resistance	Probabilistic sensitivity analysis was run within model across over 10,000 model runs • Beta distributions used for costs • Gamma distributions used for rates Prevalence of SF medicines was varied with gamma distribution	Latin hypercube sampling was conducted on 79 input parameters. CFR was chosen is important parameter
Main assumptions	Assumed global prevalence of SF medicines at 1%, 5%, and 10%; did not use individual country or regional data Assumed childhood pneumonia deaths result only from severe pneumonia Risks of 2 and 4 times the CFR were assumed to reflect the consequences of taking a substandard or falsified antibiotics for childhood pneumonia	Assumed SF antimalarials have diminished effectiveness based on API and being in a low or high transmission region. Low transmission: Assumed that effectiveness is reduced by • 30% for drugs with 75–85% API • 60% for drugs with 50–75% API • 100% (totally ineffective) for drugs with <50% API High transmission: Assumed that effectiveness is reduced by: • 25% for drugs with 75–85% API • 50% for drugs with 50–75% API • 100% (totally ineffective) for drugs with < 50% API	Use of poor-quality SP was assumed as a good proxy for the use of all poor-quality medicines—using the same rate of gametocyte carriage Assumed only SP-resistance exists Assumed half dose SP had higher gametocyte densities and longer infectivity Assumed prevalence of SF antimalarials found regionally and globally reflects antimalarials in Kenya	Assumed different prevalence scenarios for SF ACTs: • 60% genuine, 20% substandard, 20% falsified; • 85% genuine, 10% substandard, 5% falsified Assumed quality antimalarials replaced the failed batches for one month before returning to baseline level of SF Assumed one device per every 10 pharmacies in malaria endemic districts, varied in sensitivity analysis.	Assumed SF antimalarials have diminished effectiveness based on API% Assumed effectiveness is reduced by • 25% for drugs with 75–85% API • 50% for drugs with 50–75% API • 100% (totally ineffective) for drugs with < 50% API Assumed that breakdown of API percentage represents national level Assumed children who are not cured may seek care again the next week Assumed children facing a stockout may try to find medicine at another location	Assumed use of SF amoxicillin results in twice the CFR Assumed removal efforts of batches identified as SF are 100% effective Assumed 80% of pediatric pneumonia cases are prescribed with and use amoxicillin	Assumed SF antimalarials increase the CFR of malaria. Assumed SF antimalarials are only received by patients seeking care at private practices. Assumed patients cannot distinguish between SF and good-quality antimalarials (e.g., the prevalence of SF antimalarials at private facilities = the amount taken by patients)

ACT = artemisinin-based combination therapy; AL = arthemeter-lumefantrine; ALRI = acute lower respiratory infections; API = active pharmaceutical ingredient; CFR = case fatality rate; DALY = disability-adjusted life year; DHS = demographic and health survey; DRC = the Democratic Republic of the Congo; ESTEEM = Examining Screening Technologies with Economic Evaluations for Medicines model; FDD = Food and Drug Department; MIS = malaria indicators survey; MICS = UNICEF Multiple Indicator Cluster Survey; SAFARI = Substandard and Falsified Antimalarial Research Impact model; SF = substandard and falsified; SP = sulfadoxine-pyrimethamine; WHO CHOICE = World Health Organization’s Choosing Interventions That Are Cost-Effective program.

### Modeling the burden of substandard and falsified medicines.

The only global estimate came from a decision-tree model developed by the University of Edinburg and the WHO (2017), which provided approximate estimates of childhood deaths related to substandard or falsified antibiotics used for treatment of pneumonia.^1^ Starting from global estimates of childhood pneumonia mortality derived from a large burden of disease study,^13^^,^^14^ the authors calculated the number of pediatric pneumonia deaths attributable to poor-quality antibiotics. This model assumed the likely outcome of using substandard or falsified medicines was a 2-fold increase in the case fatality rate for a less effective active ingredient, or a 4-fold increase in the case fatality rate for a totally inactive antibiotic. Their estimated burden of substandard and falsified antibiotics ranged from 8,688 excess pediatric pneumonia deaths given a 1% global prevalence of substandard and falsified antibiotics to 72,430 given a 10% global prevalence.^1^

Two models examined the regional impact of substandard and falsified antimalarials in sub-Saharan Africa. The London School of Hygiene and Tropical Medicine and the WHO (2017) developed a decision tree model to simulate the infection, care seeking, and outcomes of utilizing antimalarials at varying levels of quality.^1^ The model assumed a reduction in antimalarial effectiveness based on the amount of the active pharmaceutical ingredient. The model considered that not all malaria cases will receive antimalarials due to care-seeking patterns and prescribing variations and that adherence also influences medicine effectiveness. The authors estimated that substandard and falsified antimalarials were responsible for 2.1% to 4.9% of total malaria deaths and $10.4 to $44.7 million in direct economic costs of care in sub-Saharan Africa, depending on estimates of malaria cases.^1^ Univariate sensitivity analysis found that the probability of progressing to severe malaria, probability of receiving further inpatient care after severe illness, and the cost of outpatient care had the greatest impact on results.

On the other hand, Renschler et al. (2015) estimated that poor-quality antimalarials were responsible for 122,350 (interquartile range: 91,577–154,736) pediatric malaria deaths annually across 39 sub-Saharan African countries.^4^ The model used data on the prevalence of substandard and falsified antimalarials and estimates of under-five care seeking for malaria from the literature. To account for uncertainty, this analysis used statistical modeling using Latin hypercube sampling to vary 79 input parameters, providing a range of possible outcomes. The case fatality rate used to estimate the impact of substandard and falsified antimalarials in each of the 10,000 runs directly affected the number of child deaths due to substandard or falsified antimalarials.

One agent-based model has been used to derive national estimates of the impact of poor-quality antimalarials. The Substandard and Falsified Antimalarial Research Impact (SAFARI) model has been applied in five countries to date: Benin, the Democratic Republic of the Congo, Nigeria, Uganda, and Zambia.^5^^–^^9^^,^^15^ The model incorporated demographic characteristics of a country population, which guided agents’ likelihood of being infected with malaria and care-seeking behaviors. Similar to the WHO 2017 malaria model, SAFARI assumed that antimalarial effectiveness was diminished based on the amount of API consumed. The model tracked substandard and falsified antimalarial treatments consumed, severe cases and hospitalizations, deaths, disability, costs to patients and public facilities that provide care, productivity losses of caregiver time, and productivity losses due to premature death and life lived with disability. Using the SAFARI model, the impact of substandard and falsified medicines was estimated ranging from 213 deaths or 8.1% of malaria deaths in Zambia to 12,300 deaths and $892 million in economic impact in Nigeria.^8^^,^^9^ The burden of poor-quality antimalarials weighed more heavily on poor and rural populations, furthering health inequities in Uganda.^7^

### Modeling the cost-effectiveness of medicine quality screening technologies.

Two country-level models have been developed to evaluate the cost-effectiveness of postmarket surveillance technologies to stop the circulation of substandard and falsified medicines. The Evaluating Screening Technologies for Economic Evaluation Model (ESTEEM) is an agent-based model used to simulate postmarket surveillance strategies.^10^^,^^16^ Medicines were simulated as agents and classified as quality-assured, substandard, or falsified. The model simulated sampling, screening, confirmatory testing, and removal of failed medicines from the market. The model compared use of screening technologies that could lessen the need for confirmatory testing and speed up removal of substandard and falsified medicines from the market. ESTEEM estimated the number of substandard and falsified treatments averted, deaths averted, costs of care saved, and productivity losses averted, and compared them with added costs of screening and testing medicine quality. Results from a case study in Kenya showed that faster reductions in the prevalence of substandard and falsified antibiotics can have a substantial impact on health and economic outcomes.^10^

Another model examined the cost-effectiveness of six screening technologies to identify and remove poor-quality artemisinin combination therapies (ACTs) in Laos.^11^ Using a decision tree structure, they modeled the cost-effectiveness of each screening device compared with using visual inspection in hypothetical scenarios of high and low prevalence of poor-quality ACTs. All devices were cost-effective in the high prevalence scenario, with the near infrared spectrometer (NIR-S-G1) being the most cost-effective in each scenario.

### Modeling the impact of medicine quality on antimicrobial resistance.

Only one model estimated the health impact of substandard and falsified medicines on drug resistance. Brock et al. (2017) modeled the transmission dynamics of resistant infections using mathematical models of humans and mosquitos.^12^ The model incorporated patient care-seeking, medicine use, and prevalence of substandard and falsified medicines from the literature. Half doses of sulfadoxine/pyrimethamine (SP) were used to represent poor-quality antimalarials, which resulted in longer duration of infectivity and higher malaria transmission. They found that given widespread SP resistance in Kenya, using poor-quality antimalarials increased the number of malaria cases overall (558.6% to 776.9% increase in cases) as well as the number of SP resistant infections. The authors simulated that using only quality-assured ACTs resulted in a 72.7% decrease in cases.^12^ One-way sensitivity analysis showed mosquito parameters (such as ratio of mosquitos to humans, rate of mosquito maturity, probability of sporozoite transmission) to be especially important, as well as the rate of gametocyte clearance of infections treated with good quality ACTs.

## DISCUSSION

We identified clear gaps in existing modeling literature on substandard and falsified medicines. Although the impact of substandard and falsified products is relevant for most classes of medicines, only antibiotics and antimalarials were represented in the modeling literature. A recent meta-analysis found that uterotonics, antihypertensives, and antiinflammatories, among others, failed quality tests at high rates,^17^ but estimates of the burden caused by those medicines are not yet available. In addition, we found that only countries in sub-Saharan Africa or the overall region without country-specific breakdowns were modeled. To tailor specific interventions, decision-makers require greater evidence relevant to various medicines and populations at risk.

There were commonalities across models, especially in the flow and types of inputs used. Each model began with the overall burden of disease, using either the number of cases or deaths for the country, region, or world or disease incidence. Care-seeking options were always considered, which influenced the type of medicines consumed and their quality. Finally, the outcomes depended on the quality of medicine, where simulated ineffectiveness resulted in more severe disease, further treatment, longer infectivity, or a higher probability of death. In models that included costs, we saw that the economic burden of substandard and falsified medicines fell on patients and the health care system to pay for additional costs of care, and also on society in terms of productivity losses incurred.

Two key assumptions made in each of these models indicate areas for future research and stakeholder collaboration. First, the true prevalence of substandard and falsified medicines is not known for the scope that these models assess, and literature to inform this parameter is limited. Although reports on medicine quality are increasing, the data are not always generalizable across populations. Strengthening medicines regulatory authorities to be able to regularly collect and share medicine quality data is one way to provide stronger evidence and engage stakeholders in impact modeling. The second assumption that warrants further consideration is the clinical implications of using substandard and falsified medicines. This parameter will likely vary for the medicine type as well as the quality level (substandard, falsified, or quality-assured) and has rarely been studied. The models must make assumptions about substandard and falsified medicines leading to retreatment or an increased probability of death. Gaining validation from experts, including pharmacologists with expertise in pharmacokinetics and pharmacodynamics, on the clinical burden of using substandard and falsified medicines is vital for model precision. The uncertainty in these assumptions underscores the importance of rigorous sensitivity analysis in modeling the impact of substandard and falsified medicines. Despite such uncertainty, the scale of the problem and the need for evidence by decision-makers warrant further use and development of models in this area.

An understanding of the limitations of these models is important as decision-makers look to use such evidence to inform investments towards improving medicine quality. One of the greatest limitations is the lack of evidence on the costs and effectiveness of interventions to improve medicine quality because these models focus primarily on the burden of substandard and falsified medicines rather than demonstrate the value of specific interventions. Current models do not incorporate a detailed simulation of the supply chain to identify bottlenecks or demonstrate the impact of specific policies, regulations, or postmarket surveillance measures. Greater evidence is needed across medicine quality interventions throughout the medicine supply chain to model their return on investment. Another significant limitation is the generalizability of model findings across settings and the level of heterogeneity incorporated in models to examine the impact on subpopulations. Although existing evidence offers a helpful guide, careful deliberations may be needed to interpret model findings in different settings or for specific populations.

This review is limited by search parameters and databases screened. However, we believe we have included the most relevant models that are representative of the goals and methods used in modeling the impact of substandard and falsified medicines. Although the characteristics of each model are not listed in exhaustive detail, we summarized what we found to be the most important mechanisms, data, and modeling techniques. This review identified areas for future research to better estimate the health and economic impacts of substandard and falsified medicines and make a stronger case for solutions.

## CONCLUSION

Every person has the right to expect that a medical product works as intended.^18^ Medicine quality assurance is essential for countries to reach Universal Health Coverage (UHC) goals.^19^^,^^20^ Efforts to ensure medicine quality is even more critical during the COVID-19 pandemic as regulatory and supply chain systems have been disrupted.^21^ Simulation models can provide estimates of the impact of substandard and falsified medicines that NMRAs and other stakeholders can use to advocate for needed resources to strengthen medicine quality assurance systems. To ensure that these modeled estimates are actionable, modeling efforts should be extended to include more medicines and provide country- and population-specific data.
